# Reirradiation combined with hyperthermia in recurrent breast cancer results in a worthwhile local palliation

**DOI:** 10.1038/sj.bjc.6690075

**Published:** 1999-02

**Authors:** J van der Zee, B van der Holt, P J M Rietveld, P A Helle, A J Wijnmaalen, W L J van Putten, G C van Rhoon

**Affiliations:** Department of Radiation Oncology, Subdivision of Hyperthermia, University Hospital Rotterdam, Daniel den Hoed Cancer Center, Rotterdam, The Netherlands; Department of Statistics, University Hospital Rotterdam, Daniel den Hoed Cancer Center, Rotterdam, The Netherlands; Department of Radiation Oncology, Medisch Spectrum Twente, Enschede, The Netherlands; Department of Radiation Oncology, University Hospital Rotterdam, Daniel den Hoed Cancer Center, Rotterdam, The Netherlands

**Keywords:** breast cancer, reirradiation, hyperthermia, local tumour control, palliation

## Abstract

Both experimental and clinical research have shown that hyperthermia (HT) gives valuable additional effects when applied in combination with radiotherapy (RT). The purpose of this study was evaluation of results in patients with recurrent breast cancer, treated at the Daniel den Hoed Cancer Center (DHCC) with reirradiation (re-RT; eight fractions of 4 Gy twice weekly) combined with HT. All 134 patients for whom such treatment was planned were included in the analysis. The complete response rate in 119 patients with macroscopic tumour was 71%. Including the 15 patients with microscopic disease, the local control rate was 73%. The median duration of local control was 32 months, and toxicity was acceptable. The complete response (CR) rate was higher, and the toxicity was less with the later developed 433-MHz HT technique compared with the 2450-MHz technique used initially. With this relatively well-tolerated treatment, palliation by local tumour control of a worthwhile duration is achieved in the majority of patients. The technique used for hyperthermia appeared to influence the achieved results. The value of HT in addition to this re-RT schedule has been confirmed by a prospective randomized trial in a similar patient group. In The Netherlands, this combined treatment is offered as standard to patients with breast cancer recurring in previously irradiated areas. *© 1999 Cancer Research Campaign*


*British Journal of Cancer* (1999) **79**(3/4), 483–490
© 1999 Cancer Research Campaign
Article no. bjoc.1998.0075

### Reirradiation combined with hyperthermia in recurrent breast cancer results in a worthwhile local palliation

**J van der Zee[1], B van der Holt[2], PJM Rietveld[1], PA Helle[3], AJ Wijnmaalen[4], WLJ van Putten[2] and GC van Rhoon[1]**

Department of Radiation Oncology, [1]Subdivision of Hyperthermia, and [2]Department of Statistics, University Hospital Rotterdam, Daniel den Hoed Cancer Center, Rotterdam, The Netherlands; and [3]Department of Radiation Oncology, Medisch Spectrum Twente, Enschede, The Netherlands; [4]Department of Radiation Oncology, University Hospital Rotterdam, Daniel den Hoed Cancer Center, Rotterdam, The Netherlands

**Summary** Both experimental and clinical research have shown that hyperthermia (HT) gives valuable additional effects when applied in combination with radiotherapy (RT). The purpose of this study was evaluation of results in patients with recurrent breast cancer, treated at the Daniel den Hoed Cancer Center (DHCC) with reirradiation (re-RT; eight fractions of 4 Gy twice weekly) combined with HT. All 134 patients for whom such treatment was planned were included in the analysis. The complete response rate in 119 patients with macroscopic tumour was 71%. Including the 15 patients with microscopic disease, the local control rate was 73%. The median duration of local control was 32 months, and toxicity was acceptable. The complete response (CR) rate was higher, and the toxicity was less with the later developed 433-MHz HT technique compared with the 2450-MHz technique used initially. With this relatively well-tolerated treatment, palliation by local tumour control of a worthwhile duration is achieved in the majority of patients. The technique used for hyperthermia appeared to influence the achieved results. The value of HT in addition to this re-RT schedule has been confirmed by a prospective randomized trial in a similar patient group. In The Netherlands, this combined treatment is offered as standard to patients with breast cancer recurring in previously irradiated areas.

**Keywords:** breast cancer; reirradiation; hyperthermia; local tumour control; palliation

### The clinical problem

Local regional recurrences of breast cancer may be the cause of severe suffering when uncontrolled, without being life-threatening at short term. Symptoms such as ulceration, bleeding and severe pain have been seen in 62% of the patients with recurrent breast cancer referred for radiotherapy (RT) (Bedwinek et al, 1981*a*). Furthermore, watching a growing tumour at the surface of the body is a stressful experience to the patient. The survival of patients with a locoregional recurrence appears not to be related to any local treatment; in the majority of patients, distant metastasis is identified after a median follow-up time of less than 12–30 months. Nevertheless, the median survival time in this patient group may vary from 12 to 53 months, depending mainly on tumour characteristics at the time of first diagnosis, with a 5-year survival rate of 22–50% (Bedwinek et al, 1981*b*; Toonkel et al, 1983; Patanaphan et al, 1984; Aberizk et al, 1986; Deutsch et al, 1986; Hietanen et al, 1986; Stadler and Kogelnik, 1987; Blanco et al, 1990; Schwaibold et al, 1991; Halverson et al, 1992). The financial cost of treating patients with local regional breast cancer recurrences has been estimated to be around Australian $533 per month by Hurley et al (1992). Therefore, application of a treatment which can result in long-term local tumour control would be worthwhile from the perspectives of both the patient and the health care system.

*Received 20 June 1997*
*Revised 27 April 1998*
*Accepted 15 June 1998*

*Correspondence to:* J van der Zee, Department of Radiation Oncology, Subdivision of Hyperthermia, University Hospital Rotterdam, Daniel den Hoed Cancer Center, Groene Hilledijk 301, 3075 EA Rotterdam, The Netherlands

In the case of a recurrent tumour within a previously irradiated area, the chance of achieving local control by either RT or chemotherapy is reduced (Okunieff et al, 1991). The radiation dose that can be given without a high risk of unacceptable toxicity is lower than considered adequate (Bedwinek et al, 1981a; Halverson et al, 1990; Withers et al, 1995). This poor prognosis led to the evaluation of combining re-RT and local hyperthermia (HT) in this patient group at the DHCC.

Experimental research has shown that HT is an effective cell-killing agent especially to cells in a hypoxic, nutrient deprived and low pH environment, conditions which are specifically found in malignant tumours. The combination of RT with HT should result in at least a complementary tumoricidal effect, if not a supra-additive effect (Field, 1990; Raaphorst, 1990). The existing clinical data appear to confirm the findings from experimental research. Recently, the therapeutic gain by HT in addition to RT, has been documented by randomized comparative studies in various tumour types (Valdagni et al, 1988; Overgaard et al, 1995; International Collaborative Hyperthermia Group, 1996; van der Zee et al, 1996).

At the DHCC, both treatment modalities underwent changes during the period since the combination was first applied clinically. The total re-RT dose gradually increased from about 20 to 32 Gy. The treatment schedule of eight fractions of 4 Gy, twice weekly, was first applied in 1981, and, after apparent effectiveness and tolerance (van der Zee et al, 1988), this became the protocol in 1988. Hyperthermia technique was gradually improved over the years.

### MATERIALS AND METHODS

### Patients and tumours

All 134 patients with recurrent adenocarcinoma of the breast, for whom reirradiation with eight fractions of 4 Gy combined with HT

**Table 1** Patient and tumour characteristics in relation to treatment technique

| | **433 MHz *n* = 107** | | **2450 MHz *n* = 27** | |
|---|---|---|---|---|
| | **Median (range) s.d.[a]** | **Patients** | **Median (range) s.d.[a]** | **Patients** |
| Disease-free interval from start of first treatment to first relapse (months) | 23 (1–158) 27 | | 15 (2–168) 25 | |
| Number of previously given kinds of chemotherapy | | | | |
| 0 | | 57 | | 9 |
| 1 | | 33 | | 13 |
| 2 or 3 | | 17 | | 5 |
| Number of previously given kinds of hormonal therapy | | | | |
| 0 | | 54 | | 10 |
| 1 | | 23 | | 8 |
| ≥2 | | 30 | | 9 |
| Number of previous surgical procedures at the same location | | | | |
| 0 | | 7 | | 1 |
| 1 | | 49 | | 16 |
| ≥2 | | 51 | | 10 |
| Dose of radiotherapy given previously (Gy) | 45 (20–66) 6 | | 45 (15–58) 10 | |
| Macroscopic tumour | | | | |
| ≤3 cm | | 38 | | 11 |
| >3 cm | | 57 | | 13 |
| Ulcerating tumour | | 24 | | 6 |
| Tumour histology: grade of differentiation | | | | |
| Good | | 1 | | 0 |
| Moderate | | 18 | | 1 |
| Poor | | 53 | | 12 |
| Undifferentiated | | 14 | | 7 |
| Unknown | | 21 | | 7 |
| Number of lesions | | | | |
| Single | | 42 | | 9 |
| 2 | | 19 | | 8 |
| 3–9 or more | | 46 | | 10 |
| Haemoglobin at time of treatment (mmol $l^{-1}$) | 8.3  0.7 | | 8.3  0.7 | |
| Tumour outside treatment volume | | 41 | | 10 |
| Macroscopic tumours only: | | | | |
| Tumour volume ($cm^3$) | 11 (1–868) 166 | | 6 (1–777) 39 | |
| Tumour maximum diameter (cm) | 4.9 (5–300) 6.0 | | 4.7 (6–175) 4.8 | |
| Maximum depth (cm) | 2.0 (1–9) 1.6 | | 2.0 (1–5) 0.7 | |
| Continuation of hormonal therapy during local treatment | | 15 | | 5 |

[a]s.d., standard deviation.

was planned between January 1981 and May 1992 for a total number of 143 fields, are included in this evaluation. Only the first treated field in each patient was included, leaving a total number of 134 fields in 134 patients to be analysed. Six of these patients had been included in the randomized study reported by the International Collaborative Hyperthermia Group (1996).

Patients were selected for re-RT plus HT on the following criteria: recurrent tumour, inoperable ($n = 119$) or after microscopically incomplete excision ($n = 15$); and systemic therapy was either inadequate to control the local regional tumour, or was deemed inappropriate, in the absence of (symptomatic) systemic disease.

At the time of treatment, patients were aged 28–82 years, with a median of 58 years. Performance status was generally good, with WHO scores 0 or 1 in 132 patients and 2–4 in two patients. Distant metastasis was present at the start of treatment in 38% of the patients. Seventy per cent of the patients had been treated with hormonal and/or chemotherapy in the past. Previous RT to the same area had been given 4 months to 17 years (median 41 months) before the re-RT plus HT treatment. Tumour localization was on the chest wall in 130 patients. Patient and tumour characteristics known to have prognostic value are given in Table 1, in relation to the HT technique used for treatment. In case of multiple lesions, the maximum diameter and the volume of the largest lesion was used in the evaluation. Tumour volume was calculated according to the formula $1/6\pi a*b*c$, in which $a$ and $b$ are the largest diameters measured by calipers and $c$ is the maximum extension in depth estimated by palpation and, in some cases, established by ultrasound or computerized tomography (CT) scan.

*© Cancer Research Campaign 1999*

**Table 2** Treatment characteristics

| | *n* | Range | Median (s.d.[a]) |
|---|---|---|---|
| Total re-RT dose | | | |
| <32 (12–28) Gy | 4 | | |
| =32 Gy | 129 | | |
| =36 Gy | 1 | | |
| Size of radiation field (cm$^2$) | | 30–875 | 248 (184) |
| Size of hyperthermia field (cm$^2$) | | 64–800 | 300 (155) |
| Number of HT applicator set-ups | | | |
| 1 | 101 | | |
| 2 | 7 | | |
| 3 | 2 | | |
| 4 | 2 | | |
| Received eight HT treatments | 123 | | |
| Total duration of all HT treatments (min) | | | 480 (55) |
| Number of thermometry points [median (range)]: | **433 MHz** | | **2450 MHz** |
| In tumour tissue | 5.4 (1–20) | | 2.6 (1–10) |
| In normal tissue | 13.9 (1–44) | | 2.9 (1–11) |
| HT dose parameters [median (s.d.[a])]: | **433 MHz** | | **2450 MHz** |
| $Tmaxmax_{mean}$ in tumour (°C) | 43.4 (1.3) | | 42.2 (1.4) |
| $T90_{best}$ in tumour (°C) | 40.1 (1.2) | | 40.6 (1.7) |
| $Tmaxmax_{mean}$ in normal tissue (°C) | 43.8 (1.0) | | 42.2 (1.2) |
| $T90_{best}$ in normal tissue (°C) | 39.2 (1.1) | | 39.9 (1.2) |
| Tumour tissue T90 >40°C (min) | 61 (118) | | 118 (137) |
| Normal tissue T90 >40°C (min) | 0 (55) | | 48 (101) |

[a]s.d., standard deviation.

### Treatment

All patients were treated with the same radiation schedule of 4 Gy twice weekly, each fraction followed by 1 h HT. The time interval between two re-RT + HT treatments was 3–4 days, that between the re-RT fraction and HT session was an average of 40 min. A summary of treatment characteristics is given in Table 2.

### *Radiotherapy*

The planned treatment of eight fractions of 4 Gy was applied to 129 patients. Four patients received a lower total dose, of 12–28 Gy, as their treatment was terminated because of general deterioration by rapid progression of systemic disease. In one patient, the treatment was interrupted because of development of a urinary tract infection, and a ninth fraction was given to compensate for the delay. Radiation techniques included electrons ($n = 107$), photons ($n = 15$), a combination ($n = 9$) or orthovoltage ($n = 2$) (unknown in one) depending on the tumour location and depth. The radiation field was chosen with a margin of at least 2 cm around the macroscopic tumour.

### *Hyperthermia*

Seven patients received less HT treatments than RT fractions. Four patients received seven instead of eight HT treatments because of logistics. In two patients, the treated area needed four HT applicator set-ups to cover the whole field, therefore only half of the field was treated during each session. In the one patient receiving nine fractions of RT, six HT treatments were applied. Standard treatment duration was 60 min with power on. For delivery of HT, the 2450-MHz technique was used in 27 patients (1981–86) and the 433-MHz technique in 107 patients (since 1986). The aim of the treatment was to achieve the highest tumour temperatures. Power input was limited by the temperatures measured within the tumour periphery (maximum 44°C was allowed at ≤1 cm distance from normal tissue) and the normal tissue (maximum 43°C during the first 30 min, 44°C during the second 30 min), and by power-related pain expressed by the patient at a site without thermometry. The hyperthermia field size is defined as the sum of the aperture areas of the applicators used.

### *2450 MHz technique*

Custom-built air-filled waveguide applicators, with aperture sizes of $8 \times 4$ and $8 \times 6$ cm$^2$, were used in various combinations. Up to four applicators were coupled to one power supply, without the possibility to control power supply to the individual applicators. A maximum of eight applicators could be used at the same time. Surface cooling, when necessary, was performed by directing air currents under the applicators. Interstitial thermometry was performed by thermocouples, using either single sensor probes within a needle or multisensor probes within a catheter. Temperatures were measured every 5 min with the power shut off.

### *433 MHz technique*

A dipole antenna was used in three patients. Custom-built water-filled waveguide applicators (Van Rhoon et al, 1998) were used since February 1985. The maximum number of applicators used simultaneously increased, over time, from two to five. Each applicator was supplied with independent power control. Until July 1987, the aim of the treatment was to heat the macroscopic tumour but, after experiencing tumour regrowth within the radiation field, outside the margin of the HT field (van der Zee et al, 1992); the applicator set-up was chosen such that the radiation field was widely covered. Surface temperature control was performed by using a perfused water bolus. Since May 1987, temperatures have

© Cancer Research Campaign 1999

**Table 3** Parameters included in the evaluation of prognostic factors

| | |
|---|---|
| Chemotherapy | Former chemotherapy: yes or no |
| Hormonal therapy | Former hormonal therapy: yes or no |
| Former local treatment | Two classes: either a maximum of one surgical treatment plus RT, or two or more surgical treatments plus RT |
| RT dose | Total (cumulative) dose of previously applied RT; continuous variable, or classes ≤42, >42–46 and ≥46 Gy |
| Number of lesions | Number of tumour lesions within the treatment volume; classes 1, 2 and ≥3 |
| Log tumour volume | Logarithm of volume of (largest) tumour lesion in $mm^3$; continuous variable, or classes ≤2, 2–4 and ≥4 |
| Tumour maximum diameter | Maximum diameter of (largest) tumour lesion; in two classes ≤3 and >3 cm |
| $Tmaxmax_{mean}$ in tumour | Continuous variable, or classes ≤42.5, >42.5–43.5 and ≥43.5°C |
| $T90_{best}$ in tumour | Continuous variable, or classes ≤39.6, >39.6–40.6, and >40.6°C |
| $Tmaxmax_{mean}$ in normal tissue | Continuous variable, or classes ≤43.3, >43.3–44.3, and >44.3°C |
| $T90_{best}$ in normal tissue | Continuous variable, or classes ≤38.8, >38.8–39.5 and >39.5°C |
| HT technique | Two classes: 433 MHz and 2450 MHz |

been measured continuously during treatments by a 24-channel scanning fibre-optic system (FT1210, Takaoka Japan), with which five multisensor probes (up to four sensors) and four single-sensor probes were available.

Two patients, both with a tumour extending to a depth of more than 5 cm, who were treated with other equipment (13.5 MHz capacative and 80 MHz radiative, respectively) were included in the 433-MHz technique group.

### Hyperthermia dose parameters

For both normal tissue and tumour tissue, 20 parameters representative of HT dose were calculated from the temperatures measured during heating. A full description of all these dose parameters is beyond the scope of this report, but will be published elsewhere. These parameters, however, appeared highly correlated. Factor analysis was used to select the two parameters, within tumour as well as normal tissue, that contain the most information. These parameters were $Tmaxmax_{mean}$ and $T90_{best}$. *Tmaxmax* is defined as the highest temperature measured for each heat session and, for this analysis, the average of these highest temperatures was selected. *T90* is calculated from the Gaussian distribution of all temperature measurements during each heat treatment, after the heating-up phase of 10 min, and represents the value above which 90% of all measurements were observed. The $T90_{best}$ is the T90 from the treatment session during which the highest value was achieved.

### End points

Response was established at the time of maximum regression which was observed 1–14 (median 2) months after start of treatment. A complete response (CR) is defined according to WHO criteria: clinically a complete tumour remission, observed twice with a time interval of at least 4 weeks. The duration of local control, in patients treated for microscopic tumour and in patients in whom a CR was achieved, was defined from the start of treatment till the first observation of progression within the treated volume, which is defined as the volume to which radiation was applied.

Acute toxicity observed can be distinguished to result from either RT or HT. Radiation-induced acute toxicity includes erythema (none, mild, moderate or severe) and moist desquamation. Thermal burns include second and third degree burns in the skin, and subcutaneous burns. Late radiation toxicity was scored for pigmentation, telangiectasis, subcutaneous fibrosis and ulceration. Only ulcerations observed without persistent or progressive tumour, and not resulting from a third degree thermal burn, were assessed as re-RT toxicity.

**Table 4** Complete tumour response and acute toxicity in relation to treatment technique

| | 433 MHz technique *n* = 107 (%) | 2450 MHz technique *n* = 27 (%) |
|---|---|---|
| Complete response (macroscopic disease only) | 74 | 58 |
| Max. tumour diameter ≤3 cm | 87 | 91 |
| Max. tumour diameter >3 cm | 65 | 31 |
| Local control (microscopic disease included) | 76 | 63 |
| Burns | | |
| No or first degree | 71 | 33 |
| Second degree | 19 | 56 |
| Third degree | 7 | 11 |
| Subcutaneous | 3 | 0 |
| Erythema | | |
| None–mild | 65 | 59 |
| Moderate–marked | 35 | 41 |
| Moist desquamation | 12 | 7 |

For both techniques, the difference in CR rate between small and larger tumours is significant ($P = 0.017$ for the 433-MHz technique and $P = 0.003$ for the 2450-MHz technique). The difference in CR rate between the two techniques is significant for the large tumours ($P = 0.024$).

### Statistical methods

The parameters included in the evaluation of prognostic factors are listed in Table 3. The evaluation of factors associated with CR was restricted to the 119 patients with macroscopic tumour. Patients treated after irradical resection of recurrent tumour were included in the analysis of local tumour control. Pearson's chi-squared test was used to determine which parameters were associated with CR rate or acute toxicity caused by the treatment. Cox regression, univariate as well as multivariate, was used to investigate which variables were associated with duration of local control.

 © Cancer Research Campaign 1999

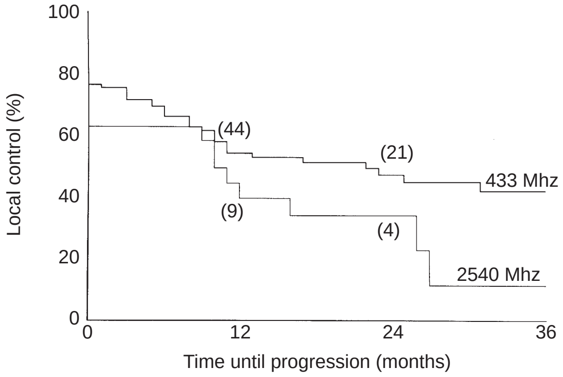

**Figure 1** Duration of local control in relation to technique used for delivery of hyperthermia: 2450 MHz or 433 MHz. Actuarial local control rates at 1 and 2 years are 44% and 33% for 2450 MHz and 54% and 47% for 433 MHz respectively (log-rank test $P = 0.05$)

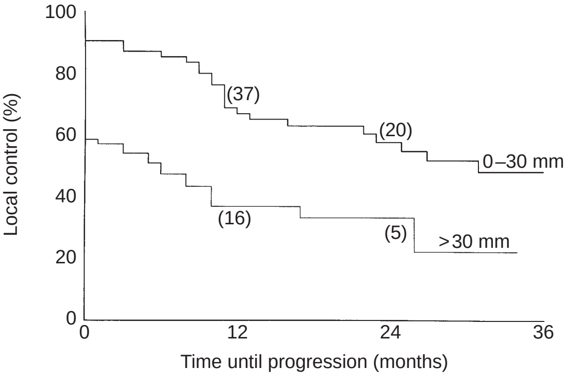

**Figure 2** Duration of local tumour control in relation to tumour size: maximum tumour diameter ≤3 cm compared with >3 cm. Actuarial local control rates at 1 and 2 years are 69% and 57% for smaller tumours and 36% and 33% for larger tumours respectively (log-rank test $P = 0.0001$)

### RESULTS

### Complete response and duration of local control

The follow-up time for all patients varied from 1 to 76 months with a median of 21 months. Five patients died within 4 weeks after the last treatment, and three patients were lost to follow-up of the locally treated area because of tumour progression outside the treatment volume for which they were treated in another hospital. These eight formally non-evaluable patients were included as non-complete responders. A CR was observed in 84 out of 119 (71%) of patients with a macroscopic tumour. The probability to achieve a CR appeared higher for patients treated with the 433-MHz technique (74%) than for those treated with the 2450-MHz technique (58%) (Table 4).

In 26% ($n$ = 35) of all patients, local tumour control was not achieved. Within the group of 99 patients with CR, or treated after microscopically incomplete resection, in-field tumour regrowth was observed in 36, after a follow-up time of 2 months to 5 years (median 11 months). The median overall survival in these patients was 20 months. Thirty-six patients have died with local tumour control after a median survival of 15 months, whereas 27 patients are still alive with local tumour control after a follow-up period of 5–76 months (median 31 months). Overall, the median duration of local control, censored for death, is 32 months.

The median overall survival time for the whole group of patients is 21 months. In the 99 patients in whom local control was achieved, the median overall survival is 31 months.

### Local tumour control in patients with microscopic disease

Fifteen patients with microscopic disease after incomplete excision were all treated with the planned re-RT of eight fractions of 4 Gy. Three of these patients were treated with the 2450-MHz technique. In all three patients, in-field tumour regrowth was observed 10–12 months after the start of treatment. In the 12 patients treated with the 433-MHz technique, in-field tumour regrowth was observed only twice, 10–13 months after the start of treatment. Three patients have died with local tumour control after 4–16 (median 10) months and seven patients are still alive with local tumour control after 16–70 (median 42) months. The difference in local control probability between the two techniques is significant ($P < 0.01$).

### Acute and late toxicity

The skin reaction resulting from re-RT was moderate to marked erythema in 48 patients, combined with moist desquamation in 15 patients. In 86 patients, the skin reaction was less severe.

Thermal burns developed in 49 patients. In three patients, all treated with the 433-MHz technique, these were located subcutaneously. There was a remarkably lower number of second and third degree thermal burns in patients treated with the 433-MHz technique (27%), compared with the patients treated with the 2450-MHz technique (67%) (Table 4). Second-degree burns generally healed within 2 months without treatment. Third-degree burns required 4–6 months of conservative treatment to heal. As these burns preferably develop at sites of limited sensitivity, they generally caused minimal symptoms.

Clinically relevant late toxicity was observed in a minority of patients. Part of the late effects of RT had been present before the start of the combined treatment, because of the previous radiation. Moderate pigmentation was observed in three patients, moderate telangiectasis in three, and subcutaneous fibrosis in 19. Ulcerations were found in 14 patients, nine of whom had this ulceration at the tumour site before treatment. Ulceration without persistent tumour was present at last follow-up in five patients. In three of these patients, this was at the site of a HT-induced burn. In two patients, the ulceration resulted from radiation damage: one in the axilla where at the start of the combined treatment severe telangiectasis was present because of previous irradiation with 50 Gy, and a second in a patient treated for an ulcerating tumour in the intact breast who had severe fibrosis because of previous RT (60 Gy). Bone necrosis, fracture or brachial plexopathy were not observed.

### Prognostic factors

#### *Influencing complete response and duration of local control*

The probability of achieving a CR decreased if patients had been previously treated with chemotherapy ($P < 0.01$) or hormonal therapy ($P < 0.02$), had larger tumour volumes ($P < 0.01$) and had larger maximum tumour diameters ($P < 0.001$). Further, the CR rate increased with higher $Tmaxmax_{mean}$ in normal tissue ($P < 0.04$). Neither the $T90_{best}$ values, for both normal ($P = 0.62$) and tumour tissue ($P = 0.30$), nor the $Tmaxmax_{mean}$ in tumour tissue ($P = 0.29$) showed an association with CR.

© Cancer Research Campaign 1999

Univariate Cox regression was used to determine factors that were of influence to continuous duration of local control. Factors influencing local control negatively were previous chemotherapy ($P = 0.01$), a higher number of lesions ($P = 0.02$), a larger tumour volume ($P = 0.01$) and a larger maximum tumour diameter ($P < 0.001$). A higher tumour $T90_{best}$ ($P = 0.02$) and a higher normal tissue $Tmaxmax_{mean}$ ($P = 0.02$) improved local control duration. It is to be noted that in the univariate Cox regression there was no significant difference in local control between patients treated with 2450 MHz and 433 MHz equipment ($P = 0.08$).

All parameters significant in the univariate regression were tested in the multivariate analysis, which further included HT technique. The multivariate Cox regression analysis showed that tumour maximum diameter, divided into two classes (≤3 cm and >3 cm), appeared to be an independent, significant ($P < 0.001$) item with regard to local control, and that 433 MHz equipment performed better than 2450 MHz equipment ($P = 0.046$). None of the other factors was significantly associated with local control. Figures 1 and 2 show the percentages of local control for 2450 MHz compared with 433 MHz equipment, and maximum tumour diameter smaller than or equal to 30 mm compared with larger than 30 mm.

### *Influencing hyperthermia damage*

The only parameter influencing risk of burns was the technique used for delivery of HT. Univariate logistic regression showed that 433 MHz treatments caused much less acute damage ($P < 0.001$) than 2450 MHz treatments. Neither the $T90_{best}$ nor the $Tmaxmax_{mean}$ thermal dose parameters for both normal and tumour tissue influenced hyperthermia-induced damage ($P$-values varying between 0.19 and 0.52). None of the evaluated parameters were associated with late damage.

### DISCUSSION

Treatment with a radiation dose of only 32 Gy in combination with hyperthermia resulted in a complete response in 71% of the patients with macroscopic tumours. With RT alone at doses of 30–40 Gy, CR rates varying from 20% to 48% have been reported for breast cancer (van der Zee and Vernon, 1996). The same RT schedule of eight fractions of 4 Gy without HT has been applied in the RTOG 81-04 study, resulting in overall 26% complete response (Perez et al, 1989). Recently, the contribution of HT to the result of the combined treatment has been confirmed by a randomized study (International Collaborative Hyperthermia Group, 1996). Within the ESHO 5-88, comparing re-RT alone (same schedule as applied in the present study) with re-RT plus HT, the CR rate after combined treatment was 78%, which was significantly higher than the 38% CR after re-RT alone. This randomized study also demonstrated that the difference in local control is durable.

We do not expect that a locally controlled chestwall recurrence will influence overall survival. Nevertheless, the absence of symptomatic local tumour can result in an improvement in quality of life (Liu et al, 1996). When a CR has been achieved, the median duration of local control was 32 months. In 27% of all patients, in-field tumour regrowth was observed after a median follow-up time of 11 months. The median overall survival time in this group of patients was 20 months, which means that the local palliation was maintained for half of the remaining life span. Twenty-seven per cent of the patients have died without local tumour regrowth, after a median follow-up time of 15 months, whereas 20% of the patients were free of local disease at last follow-up after a median follow-up time of 31 months.

Therefore, the effect of the treatment is worthwhile for the majority of patients, whereas the treatment causes limited inconvenience. The overall duration of a treatment series is 3.5–4 weeks, during which period patients come to the hospital twice weekly for around 2 h. The HT treatment is generally well tolerated. During treatment, patients were instructed to report pain immediately; in fact this 'subjective thermometry' is a very important parameter in treatment control and should not be considered a side-effect of HT. The interstitial catheters for thermometry generally do not cause relevant problems (van der Zee et al, 1987).

The side-effects of the treatment are acceptable; the HT-induced burns generally cause no pain because of their occurrence at sites of decreased sensitivity. Side-effects other than thermal burns were no different than those expected from re-RT alone, i.e. erythema and, in about 10% of the patients, moist desquamation. Clinically relevant treatment-related late toxicity was observed in only five patients, with ulceration due to either HT or RT toxicity.

The indication to offer combined treatment to patients after incomplete resection of their recurrence was similar to that for patients with macroscopic tumours: the safe re-RT dose is inadequate for tumour control. For achievement of high local control rates with elective radiation, a dose of about 50 Gy in 2-Gy fractions is required (Bedwinek et al, 1981*a*; Withers et al, 1995). Our results in this subgroup demonstrate that additional HT at an adequate level is beneficial for these patients. Although the numbers are small, the percentage of patients treated with the 433-MHz technique in whom local control was maintained is significantly higher compared with patients treated with the 2450-MHz technique. This difference cannot be explained by a better patient selection. A comparison of the time interval between the primary RT and re-RT between the 2450-MHz and 433-MHz technique groups showed no difference. Another indication of a beneficial effect of HT in microscopic disease is the previous observation of re-recurrences in five patients in which HT was applied to the macroscopic tumour only, within the re-RT-alone part of the treated area (van der Zee et al, 1992).

The results achieved with the 433-MHz technique are remarkably better than those achieved with the 2450-MHz technique. Multivariate Cox regression of prognostic parameters showed only two parameters to be associated with local control duration, i.e. tumour size and HT technique. The advantage of using 433 MHz or, in two cases, lower frequencies instead of 2450 MHz is that with the lower frequencies the penetration depth, and thereby the heated volume, is larger, which can be expected to result in an adequate temperature increase in a larger part of the tumour volume. The improvement in temperature distribution cannot be deduced from the thermal dose parameters calculated, which may be explained by the higher number of intratumour temperature measurements with the 433-MHz technique compared with the 2450-MHz technique (van der Zee et al, 1993). The improvement of results with the better heating technique in tumours with a maximum diameter of >3 cm underscores that it is important to use a technique from which one can expect adequate tissue heating. In fact, the 31% CR rate achieved in the larger (≥3 cm maximum diameter) tumours with the 2450-MHz technique is not different from the CR rates found after re-RT alone with the same treatment schedule, i.e. 26% (Perez et al, 1989) and 38% (International Collaborative Hyperthermia Group, 1996). The importance of hyperthermia treatment

 © Cancer Research Campaign 1999

technique has been previously shown by Myerson et al, (1990). The results of the study presented here have shown that there is room for further improvement. In patients treated with the 433-MHz technique, the CR rate of 65% in patients with larger tumours is still significantly lower than that for patients with smaller tumours. We speculate that when optimum HT techniques will be available for all tumours, the overall CR rate may reach a value of about 90%. The decrease in HT-related toxicity with the use of the 433-MHz technique is another welcome improvement. Although blisters and third-degree burns generally do not result in relevant clinical problems, because of the fact that these develop at sites of decreased sensitivity, it is preferable to avoid such side-effects. The most likely explanation of the lower number of burns with the 433-MHz technique is the better control of superficial temperatures by the perfused water bolus.

This study allows a few more answers to clinically relevant questions, which have to be precautious in view of the retrospective character of the analysis. In the first place, it has been suggested that local therapy for recurrent breast cancer should be accompanied by systemic therapy (Kapp, 1996). It has been shown that systemic treatment after local treatment significantly improved disease-free survival, but had no impact on overall survival (Borner et al, 1994). In our study, continuation of chemotherapy during the local treatment series was not allowed, but in 18 patients with macroscopic tumours hormonal therapy was continued. The CR rate in patients continuing hormonal therapy was (not significantly) higher than in the remaining patients: 89% compared with 67%. However, the risk of selection bias in this comparison is obvious: 75% of the patients continuing hormonal therapy had started this more than 2 months previously, indicating that the therapy had beneficial effects. These patients, therefore, may represent a subgroup with a better prognosis. Secondly, tumour extension in depth is considered a very important selection criterion for superficial hyperthermia. We now accept patients with tumours up to 4 cm maximum depth for treatment with 433 MHz. In this study, there were 13 patients with deeper tumours. The CR rate in these patients of 62% is not significantly lower than the 72% in the remaining patients, and appears higher than observed after RT alone. This may be explained by the exceptions that we have allowed to the maximum 4-cm depth criterion. These were tumours with overlying subcutaneous fat, in which less energy is absorbed than in tissues with a high water content, and spherical tumours protruding above the skin surface, in which accumulation of energy from the various applicators can be expected at depth and in which the depth may decrease during the treatment period when the tumour regresses. A third clinically important question is whether the eight fractions of 4 Gy plus HT schedule can be applied after previous RT, up to a total dose of 60 Gy or more. In our material, the CR rate of 50% in a subgroup of eight such patients seemed lower than the 76% in the remaining patients, and a persistent ulcer developed in one patient. Further, this subgroup appeared to have a relatively short remaining lifetime compared with the other patients. Nevertheless, the 50% chance to achieve a CR may be worthwhile, depending on the symptoms of local disease and survival prognosis.

### CONCLUSIONS

This study has shown that with the combination of re-RT, eight fractions of 4 Gy in 4 weeks, and HT successful palliation of local tumour recurrence of a worthwhile duration can be achieved in the majority of patients. In addition, this treatment is well tolerated with acceptable toxicity. In The Netherlands, this combined treatment is standard therapy offered to patients with locoregional recurrent breast cancer in a previously irradiated site, providing that an adequate heating equipment is available. This study has also shown that the hyperthermia treatment technique is important for clinical outcome. The development of better HT treatment techniques, enabling a more effective treatment of larger tumours, deserves further investigations.

### ACKNOWLEDGEMENTS

The skills and care of the HT technicians MP Broekmeyer-Reurink, EM Verloop-van ’t Hof and physics assistant J Stakenborg form a contributing factor to both the good patient tolerance and the treatment results which should not be underestimated.

The cooperation in this study of other radiation oncologists (formerly) at the DHCC (AD Treurniet-Donker, JJ Seldenrath, JH Meerwaldt, SK The, BA Reichgelt, WAM Mellink, AJ Subandono Tjokrowardojo, PLA van den Ende, PCM Koper, MJM van Mierlo, and B Veeze-Kuijpers) and from other centres (MFH Dielwart and JM Tabak, Zeeuws Radiotherapy Institute, Vlissingen; FH Hoekstra, Westeinde Hospital, The Hague; RJL Caspers, University Hospital, Leiden; JJ Jobsen and M van Reyn, Medisch Spectrum Twente Enschede) is highly appreciated.

The clinical work would not have been possible without the financial support by the Dutch Cancer Society, grants no. EUR 77-4, RRTI 83-4 and DDHK 93-603, the Maurits and Anna de Kock Foundation, the Nijbakker Morra Foundation, Willem H Kröger Foundation, the Foundation Bevordering Volkskracht and the Foundation Promesa, for which the authors wish to express their gratitude.

### REFERENCES

Aberizk WJ, Silver B, Henderson IC, Cady B and Harris JR (1986) The use of radiotherapy for treatment of isolated locoregional recurrence of breast cancer after mastectomy. *Cancer* **58**: 1214–1218

Bedwinek JM, Fineberg B, Lee J and Ocwieza M (1981*a*) Analysis of failures following local treatment of isolated local-regional recurrence of breast cancer. *Int J Radiat Oncol Biol Phys* **7**: 581–585

Bedwinek JM, Lee J, Fineberg B and Ocwieza M (1981*b*) Prognostic indicators in patients with isolated local-regional recurrence of breast cancer. *Cancer* **47**: 2232–2235

Blanco G, Holli K, Heikkinen M, Kallioniemi O-P and Taskinen P (1990) Prognostic factors in recurrent breast cancer: relationships to site of recurrence, disease-free interval, female sex steroid receptors, ploidy and histological malignancy grading. *Br J Cancer* **62**: 142–146

Borner M, Bacchi M, Goldhirsch A, Greiner R, Harder F, Castiglione M, Jungi WF, Thürlimann B, Cavalli F, Obrecht JP, Leyvraz S, Alberto P, Adam H, Varini M, Loehnert T, Senn HJ, Metzger U and Brunner K (1994) First isolated locoregional recurrence following mastectomy for breast cancer: results of a phase III multicenter study comparing systemic treatment with observation after excision and radiation. *J Clin Oncol* **12**: 2071–2077

Deutsch M, Parsons JA and Mittal BB (1986) Radiation therapy for local-regional recurrent breast carcinoma. *Int J Radiat Oncol Biol Phys* **12**: 2061–2065

Field SB (1990) In vivo aspects of hyperthermic oncology. In *An Introduction to the Practical Aspects of Clinical Hyperthermia*. Field SB and Hand JW (eds.), pp. 55–68. Taylor and Francis: London

Halverson KJ, Perez CA, Kuske RR, Garcia DM, Simpson JR and Fineberg B (1990) Isolated local-regional recurrence of breast cancer following mastectomy: radiotherapeutic management. *Int J Radiat Oncol Biol Phys* **19**: 851–858

Halverson KJ, Perez CA, Kuske RR, Garcia DM, Simpson JR and Fineberg B (1992) Survival following locoregional recurrence of breast cancer: univariate and multivariate analysis. *Int J Radiat Oncol Biol Phys* **23**: 285–291

© Cancer Research Campaign 1999

Hietanen P, Miettinen M and Mäkinen J (1986) Survival after first recurrence in breast cancer. *Eur J Cancer Clin Oncol* **22**: 913–919

Hurley SF, Huggins RM, Snyder RD and Bishop JF (1992) The cost of breast cancer recurrences. *Br J Cancer* **65**: 449–455

International Collaborative Hyperthermia Group (1996) Hyperthermia in the treatment of superficial localized primary and recurrent breast cancer – results from five randomized controlled trials. *Int J Radiat Oncol Biol Phys* **35**: 731–744

Kapp DS (1996) Efficacy of adjuvant hyperthermia in the treatment of superficial recurrent breast cancer: confirmation and future directions. *Int J Radiat Oncol Biol Phys* **35**: 1117–1121

Liu F-F, Bezjak A, Levin W, Cooper B, Pintilie M and Sherar MD (1996) Letter to the Editor. Assessment of palliation in women with recurrent breast cancer. *Int J Hyperthermia* **12**: 825–826

Myerson RJ, Perez CA, Emami B, Straube W, Kuske RR, Leybovich L and Von Gerichten D (1990) Tumor control in long-term survivors following superficial hyperthermia. *Int J Radiat Oncol Biol Phys* **18**: 1123–1129

Okunieff P, Urano M, Kallinowski F, Vaupel P and Neuringer LJ (1991) Tumors growing in irradiated tissue: oxygenation, metabolic state, and pH. *Int J Radiat Oncol Biol Phys* **21**: 667–673

Overgaard J, González González D, Hulshof MCCM, Arcangeli G, Dahl O, Mella O and Bentzen SM (1995) Randomised trial of hyperthermia as adjuvant to radiotherapy for recurrent or metastatic malignant melanoma. *Lancet* **345**: 540–543

Patanaphan V, Salazar OM and Poussin-Rosillo H (1984) Prognosticators in recurrent breast cancer. A 15-year experience with irradiation. *Cancer* **54**: 228–234

Perez CA, Gillespie B, Pajak T, Hornback NB, Emami B and Rubin P (1989) Quality assurance problems in clinical hyperthermia and their impact on therapeutic outcome: a report by the radiation therapy oncology group. *Int J Radiat Oncol Biol Phys* **16**: 551–558

Raaphorst GP (1990) Fundamental aspects of hyperthermic biology. In *An Introduction to the Practical Aspects of Clinical Hyperthermia*. Field SB and Hand JW (eds.), pp. 10–54. Taylor and Francis: London

Schwaibold F, Fowble BL, Solin LJ, Schultz DJ and Goodman RL (1991) The results of radiation therapy for isolated local regional recurrence after mastectomy. *Int J Radiat Oncology Biol Phys* **21**: 299–310

Stadler B and Kogelnik HD (1987) Local control and outcome of patients irradiated for isolated chest wall recurrences of breast cancer. *Radiother Oncol* **8**: 105–111

Toonkel LM, Fix I, Jacobson LH and Wallach CB (1983) The significance of local recurrence of carcinoma of the breast. *Int J Radiat Oncol Biol Phys* **9**: 33–39

Valdagni R, Amichetti M and Pani G (1988) Radical radiation alone versus radical radiation plus microwave hyperthermia for N3 (TNM-UICC) neck nodes: a prospective randomized clinical trial. *Int J Radiat Oncol Biol Phys* **15**: 13–24

Van Rhoon GC, Rietveld PJM, Van der Zee J and Van den Berg AP (1998) A 433 MHz Lucite Cone waveguide applicator for superficial hyperthermia. *Int J Hyperthermia* **14**: 13–27

Van der Zee J and Vernon CC (1996) Thermoradiotherapy for advanced and recurrent breast tumours. In *Medical Radiology. Thermoradiotherapy and Thermochemotherapy*, vol 2. Seegenschmiedt MH, Fessenden P and Vernon CC (eds.), pp. 35–48. Springer-Verlag: Berlin

Van der Zee J, Van Rhoon GC, Broekmeyer-Reurink MP and Reinhold HS (1987) The use of implanted closed-tip catheters for the introduction of thermometry probes during local hyperthermia treatment series. *Int J Hyperthermia* **3**: 337–345

Van der Zee J, Treurniet-Donker AD, The SK, Helle PA, Seldenrath JJ, Meerwaldt JH, Wijnmaalen AJ, Van den Berg AP, Van Rhoon GC, Broekmeyer-Reurink MP and Reinhold HS (1988) Low dose reirradiation in combination with hyperthermia: a palliative treatment for patients with breast cancer recurring in previously irradiated areas. *Int J Radiat Oncol Biol Phys* **15**: 1407–1413

Van der Zee J, Van Rhoon GC, Koper PCM and Van den Berg AP (1992) Clinical application and specific requirements of local hyperthermia for chest wall recurrences. *Strahlenther Onkol* **168**: 653–654

Van der Zee J, Van Rhoon GC, Verloop-van ’t Hof EM, Van der Ploeg SK, Rietveld PJM and Van den Berg AP (1993) The importance of adequate heating techniques for therapeutic outcome. In *Hyperthermic Oncology 1992*, vol II. Gerner EW and Cetas CT (eds.), pp. 349–352. Arizona Board of Regents: Tucson

Van der Zee J, González González D, Van Rhoon GC, Van Dijk JDP, Van Putten WLJ, Hart AAM, Koper PCM, De Wit GA and De Charro FTh (1996) Results of additional hyperthermia in inoperable pelvic tumours. In *Hyperthermic Oncology 1996*. Franconi C, Arcangeli G and Cavaliere R (eds.), pp. 215–217. Tor Vergata Post Graduate School of Medical Physics: Rome

Withers HR, Peters LJ and Taylor JMG (1995) Dose–response relationship for radiation therapy of subclinical disease. *Int J Radiat Oncol Biol Phys* **31**: 353–359

*© Cancer Research Campaign 1999*
